# Use of Customized 3D-Printed Titanium Augment With Tantalum Trabecular Cup for Large Acetabular Bone Defects in Revision Total Hip Arthroplasty: A Midterm Follow-Up Study

**DOI:** 10.3389/fbioe.2022.900905

**Published:** 2022-06-01

**Authors:** Keyu Kong, Chen Zhao, Yongyun Chang, Hua Qiao, Yi Hu, Huiwu Li, Jingwei Zhang

**Affiliations:** Shanghai Key Laboratory of Orthopaedic Implants, Department of Orthopaedic Surgery, Shanghai Ninth People’s Hospital, Shanghai Jiaotong University School of Medicine, Shanghai, China

**Keywords:** revision hip arthroplasty, 3D-printed titanium augment, trabecular tantalum cup, bone defects, rapid prototype

## Abstract

**Aims:** In revision total hip arthroplasty (THA), large acetabular bone defects pose challenges for surgeons. Recently, wide application of trabecular tantalum, which has outstanding biocompatibility and mechanical properties, and the development of three-dimensional (3D) printing have led to the introduction of new schemes for acetabular reconstruction. However, few studies have focused on the treatment of bone defects with customized 3D-printed titanium augments combined with tantalum trabecular cup. Thus, we aimed to evaluate the effect of this therapy in patients who underwent revision THAs.

**Patients and Methods:** We included 23 patients with Paprosky type III acetabular bone defects who underwent revision THA between January 2013 and June 2019. The preoperative hip rotation center and functional score were compared with those at 2–7 years (average 4.7 years) postoperatively to evaluate the midterm prognosis of our treatment choice.

**Results:** Postoperatively, the rotation centres of all hips were comparable with those of the contralateral hips. Hip function improved with average Harris Hip Score improved from 33.5 (22.7–40.2) to 86.1 (73.5–95.6) and average Oxford Hip Score improved from 8.3 (0–14) to 38.8 (35–48) during follow-up. One dislocation, which occurred due to extreme hip flexion within 6 weeks, was treated with closed reduction, and no recurrent dislocation occurred. No nerve injury, infection, aseptic loosening, or osteolysis were observed and no re-revision was performed in any patient.

**Conclusion:** Satisfactory midterm outcomes were obtained with 3D-printed titanium augment combined with tantalum cup for the treatment of acetabular defects in revision THA. Changes in the Harris Hip Score and Oxford Hip Score suggested a significant improvement in hip function.

## Introduction

Currently, the number of revision hip arthroplasties is increasing rapidly (Schwartz et al., 2016; Gwam et al., 2017). Acetabulum reconstruction by filling the bone defect, initial stability of the prosthesis, and restoration of the hip rotation center are the main objectives of revision surgery (Johnston et al., 1979). In patients with severe bone defects, inadequate and poor-quality residual bone poses challenges to surgeons in terms of providing effective support for prosthesis reconstruction (Knight et al., 1993). Currently, reconstruction of acetabular bone defects are reconstructed using structural allografts, antiprotrusion cages, augments, bone impaction grafting with metal meshes, and customized triflange component (Jain et al., 2014; Baauw et al., 2016). Augments can effectively fill acetabular bone defects, and satisfactory long-term results have been reported (Del Gaizo et al., 2012; Xiao et al., 2021).

Recently, customized implants fabricated using three-dimensional (3D) printing have been applied in clinical practice (Geng et al., 2020; Zampelis and Flivik, 2020). In patients with complex and irregular acetabular defects, 3D printing and rapid prototyping (RP) can be used to fabricate customized prostheses. Since computed tomography (CT) and design of augments can be completed in an outpatient setting, patients’ hospital stay and costs are reduced (Mao et al., 2015; Li et al., 2016).

Tantalum is an ideal implant material with excellent histocompatibility (Bobyn et al., 2004; Levine B. et al., 2006; Weeden and Schmidt, 2007), and previous studies have proven that tantalum does not induce rejection and can effectively promote bone integration (Bobyn et al., 1999; Mrosek et al., 2009; Wei et al., 2016). Additionally, tantalum has excellent mechanical properties including a high friction coefficient. Trabecular tantalum has an elastic modulus similar to that of subchondral bone, which is conducive to the adhesion and growth of osteoblasts, and promotes long-term osteogenesis (Bobyn et al., 1980; Findlay et al., 2004; Meneghini et al., 2010; Wei et al., 2016).

Although 3D printing, RP, and tantalum have been used in clinical practice for many years, there are no reports on the combined application of a tantalum trabecular cup and a 3D-printed titanium augment to the best of our knowledge. Therefore, we retrospectively evaluated whether combined therapy could: 1. effectively reconstruct severe acetabular defects and improve function; 2. restore the hip rotation center; and 3. reduce the occurrence of complications such as aseptic loosening, osteolysis and infection during midterm follow-up.

## Methods

### Study Participants

This study was approved by the Ethics Committee of our institution. The medical records of all patients who underwent revision total hip arthroplasty (THA) between January 2013 and June 2019 were retrospectively reviewed. Patients with severe acetabular defects in whom difficulty existted in providing effective support with off-the-shelf augments and cups based on RP-assisted simulative surgery evaluation were included. The detailed evaluation process was similar to that in a previous study (Zhang et al., 2021). A total of 310 patients underwent revision THA, of whom 72 were classified as Paprosky type III. After excluding 49 patients who received other revision schemes (25 cases of cages and 24 cases of conventional augments), 23 patients who received 3D-printed titanium augments combined with tantalum trabecular cups were included. Among them, 17 and six patients were further classified as subtypes IIIA and IIIB, respectively. Demographic statistics are presented in Table 1. The Paprosky classification was proposed by surgeons having >15 years of experience in joint surgery who perform >200 arthroplasties each year (ZZ and HL). The exclusion criteria were as follows: 1. patients with mild bone defects (Paprosky types I and II), 2. patients receiving revision for infections and other causes with no acetabular defects, 3. patients receiving revision THAs without 3D-printed augments and tantalum cups, and 4. patients who refused to participate in our study.

**TABLE 1:** Patient demographics.

Demographic	Patients
Numbers of Patients (hips)	23 (23)
Gender (male/female)	11/12
Average follow-up period (range) (year)	4.7 (2–7)
Age (ys)^a^	65.9 ± 5.6
Height (cm)^a^	165 ± 9.4
Weight (kg)^a^	64 ± 10.1

a Values are expressed as the mean ± standard deviation.

### 3D-Printed Augment Design

All patients underwent pelvic CT before surgery. Pelvic CT was performed with patients in the supine position with 0.625-mm slices. All CT scans were performed in same medical imaging centre using the same parameters.

Mimics software was used to obtain a digital 3D-reconstructed model of the pelvis from the CT data. During reconstruction, doctors and engineers cooperate to identify effective bone mass to overcome influence of metal artifact and ensure the accuracy of model. Debridement and reaming were predicted and simulated during 3D-reconstruction and a life-size 3D model of the pelvis was printed using stereolithography. Installation simulation was performed on the RP by surgeons with >15 years of experience in joint surgery as mentioned above. Off-the-shelf cups and augments were preferred when they could be properly supported by the host bone. Otherwise, customized prosthesis was considered. Based on the clinical condition, two different strategies were applied. If the anteroinferior and posteroinferior acetabular bone was intact, the defect was reconstructed using a customized augment to support the acetabular cup. A customized cage or even a semi-pelvic prosthesis was considered when anteroinferior and posteroinferior acetabular structure were damaged and even a high risk of pelvic discontinuity exists.

Three points need to be considered while designing customized augments as we discussed in our related work (Li et al., 2013; Li et al., 2016):

1. Ensure reliable fixation of the augment to the host bone to fill bone defects. For a cavity defect, shape matching could be adopted to hold augment with surrounding host bone and we could strengthen fixation with screws if necessary. For an uncontained defect, it is necessary to design augment with flange and fix it with screws. The specific fixation area and location are determined based on the situation of bone defect and host bone presented in the rapid prototype.
2. Ensure effective stress conduction in load-bearing areas, which host in the posterosuperior part of acetabulum. Ideally, augment effectively fills the space between cup and host bone in load-bearing area to facilitate load-bearing stress transmission directly through augment. In such cases, rotational shear stress of cup is converted into compressive stress of augment.
3. The contact surfaces of the augment and cup must be matched with pre-planned cup size and allows for a bigger or smaller one size. Cup size may vary from preoperative plan (Wang et al., 2018) and the augment must therefore be designed to allow adjustment of the cup size.

**FIGURE 1:**
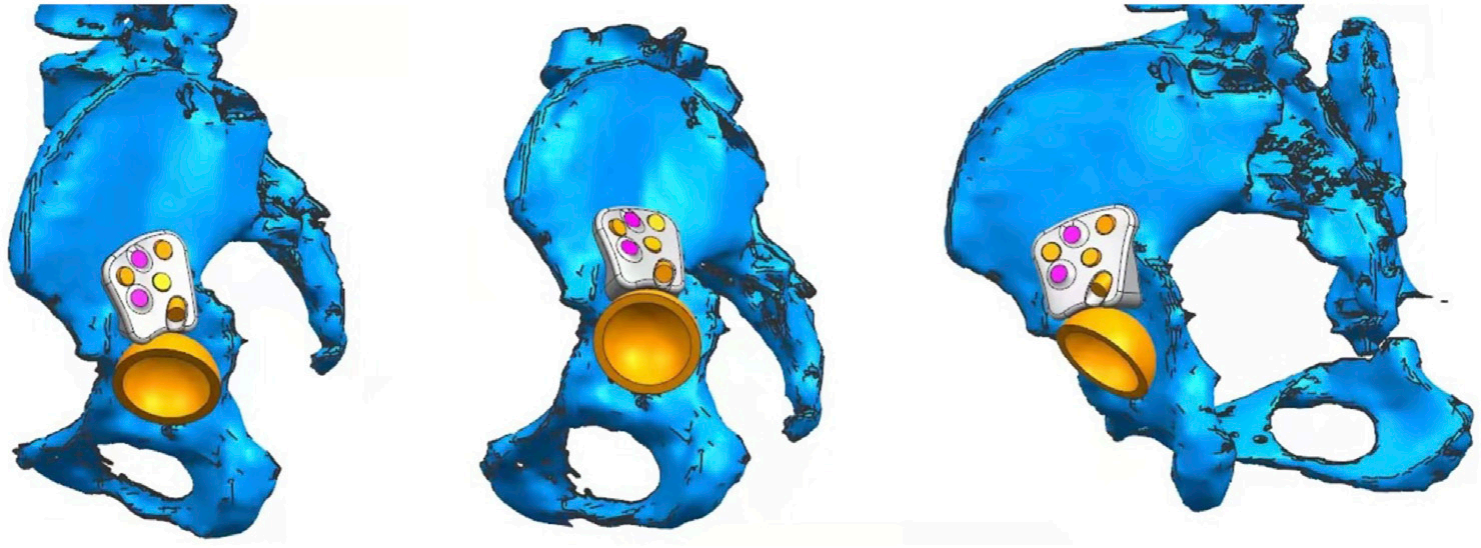
Design of customized augment from different angles. Reliable fixation and effective stress conduction were achieved between host bone and augment.

**FIGURE 2:**
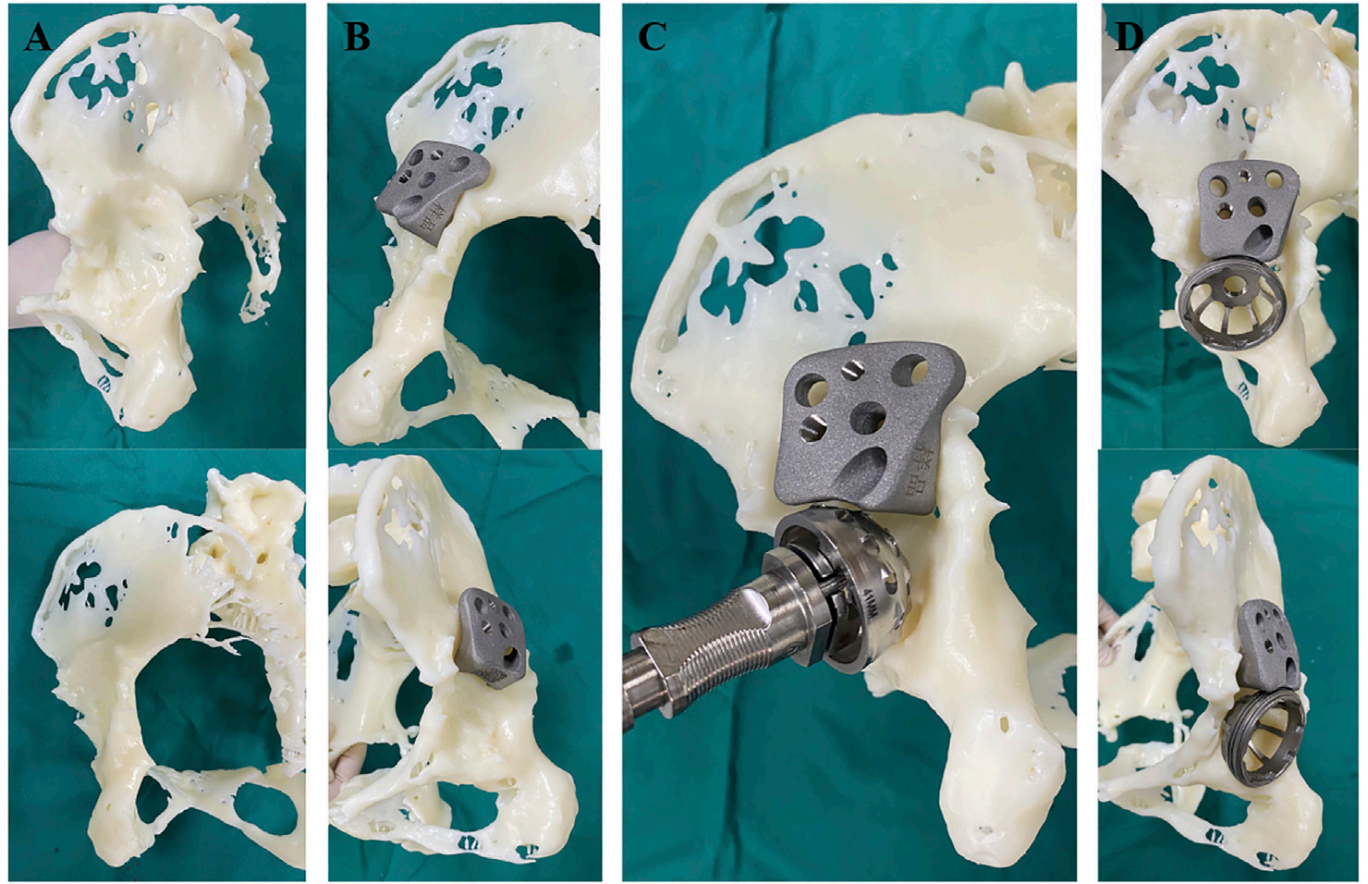
Procedure of rapid prototype-assisted evaluation. **(A)**. Reconstruction of a patient’s pelvic with rapid prototype. **(B)**. 3D-printed augment could achieve enough contact with host bone. **(C)**. Acetabulum was further filed to host acetabular cup. **(D)**. Enough contact surface was achieved between cup, augment and host bone. Acetabular defect was filled with this combined therapy.

Customized augments were printed using Ti6Al4V powder as a raw material using the selective laser melting technique. Size and shape of customized augments were determined by experienced surgeons (ZZ and HL) through simulation surgery. The printing process was completed in 1 day and printed augments were verified preoperatively using RP. The augment design is shown in Figure 1 and procedures for RP evaluation and trial installation are illustrated in Figure 2.

### Surgical Techniques

The revision procedures were performed by ZZ and HL using the posterolateral approach. During the preoperative RP trial installation, we evaluated whether the augment could effectively hold the cup together with the residual host bone; thereafter the customized augment was implanted and fixed with screws if necessary. If screw fixation was required, locking screws were used first to avoid positional changes in the augment compared to the trial installation. Bone cement was used between the augment and cup. The cup was fixed with screws after press fit, and morselized bone graft was packed to fill the remaining defect when needed.

### Postoperative Care

For the first 2–6 weeks after surgery, patients were allowed no or limited toe-touch weightbearing ambulation. After 6 weeks, patients were allowed partial weightbearing with crutches, and full weightbearing was allowed after 3 months.

### Radiographic and Clinical Outcomes

Radiographs and CT scans of all included patients before and after revision surgery were collected. Additionally, anteroposterior and lateral radiographs were taken at 3 weeks, 6 weeks, 3 months, 6 months, and 1 year after surgery and annually thereafter.

**FIGURE 3:**
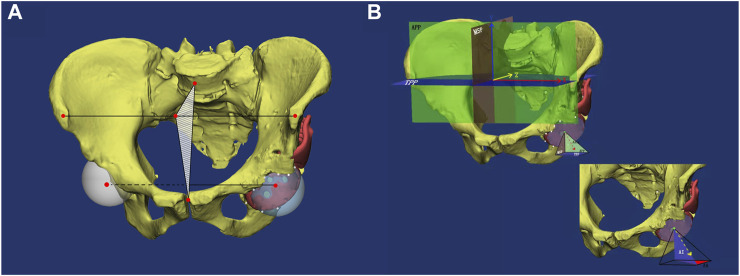
A diagram to illustrate choice of reference film, rotation center and measurement of anteversion and abduction angle. **(A)**. Midsagittal plane and reconstruction of rotation center. White points were chosen around cup surface as markers to help reconstruct rotation center (two red points in acetabulum). Midsagittal plane was determined by midpoint of bilateral anterosuperior iliac spines, center of pubic symphysis and the midpoint of fifth lumbar vertebra. **(B)**. Three reference films and the measurement of anteversion and abduction angle of cup. APP: anterior pelvic plane. MSP: midsagittal plane. TPP: transverse pelvic plane. AA: anatomic anteversion. AI: anatomic inclination.

The inclination of the cup and hip rotation center were measured on postoperative CT images as shown in Figure 3. On the prosthesis side, 5–7 points were marked on the cambered surface of the prosthesis cup to define a circular plane of orientation of the cup. Based on the circular plane and shape of cup, a concentric ball attached to the cup was reconstructed, and the centre of the ball was defined as the centre of rotation. On the contralateral side, similar approach was applied and the centre of the ball reconstructed by marking the acetabulum was defined as the centre of rotation.

### Selection of Reference Planes

The anterior plane was defined as a plane passing through the bilateral anterosuperior iliac spines and the midpoint of the pubic symphysis. The horizontal plane was defined as a plane passing through the bilateral anterosuperior iliac spines and perpendicular to the anterior plane. The midsagittal plane was defined as a plane through the midpoint of the bilateral anterosuperior iliac spines and perpendicular to the horizontal and anterior planes.

For measurement of the relative position of the centres of rotation, vertical lines were drawn from the centres of rotation on both sides to the midsagittal plane, and the difference in their lengths was calculated as the relative mediolateral relationship between the two centres of rotation; similarly, the relative anteroposterior and superoinferior relationships were defined by calculating the difference between the lengths of vertical lines perpendicular to the anterior and horizontal planes, respectively.

For measurement of the anteversion and abduction angles, a circle fitting the outer edge of the acetabular cup was marked. The anteversion and abduction angles were defined as the projection angles between the normal plane of the fitting circle and the anterior pelvic plane and horizontal plane of pelvis, respectively.

The Harris hip score and Oxford score are functional scores to assess pain, deformity, and function of the hip after surgery and are widely used to evaluate the success of revision (Harris, 1969; Murray et al., 2007). Motion range and deformity section in Harris hip score were evaluated independently by three researchers at each follow-up.

The overall survival of revision prostheses and occurrence of complications were recorded. Cup loosening was defined as described in a previous study (Li et al., 2016). Migration of prostheses was determined by comparing radiographs taken at the last follow-up with those taken immediately postoperatively. Definite loosening was defined as acetabular migration of ≥2 mm with implant rotation or screw breakage. Probable loosening was defined as a radiolucent line >1 mm through all three acetabular zones without any signs of migration, rotation, or screw breakage. Osteolysis surrounding the components was evaluated using the DeLee and Charnley and Gruen methods (DeLee and Charnley, 1976; Gruen et al., 1979). Cup loosening and osteolysis were evaluated independently by three experienced surgeons.

### Statistical Analysis

Quantitative data with normal distribution are presented as means with ranges, and categorical variables are presented as percentages. Statistical analysis was conducted using SPSS version 17.0 for windows (SPSS Inc., Chicago, IL, United States). Paired Student’s t-test was used to compare the Harris hip score and Oxford hip score. Two-sided *p* < 0.05 was considered statistically significant. Using the power analysis software PASS 16 (NCSS, LLC. Kaysville, Utah, United States), all the tests in this study with a sample size of 23 participants own a statistical power of more than 0.9 at a two-sided 5% level of significance.

## Results

The Harris hip scores before surgery and at the last follow-up were 33.5 ± 11 (22.7–40.2) and 86.1 ± 19 (73.5–95.6), respectively (*p* < 0.001). Similarly, the Oxford scores before surgery and at the last follow-up were 8.3 ± 2.6 (0–14) and 38.8 ± 1.7 (35–48), respectively (*p* < 0.001).

Compared with the contralateral side, the centre of rotation was displaced 3.7 ± 3.3 mm (0.3–7.0 mm) upward postoperatively. The absolute anteroposterior displacement was 4.1 ± 6.4 mm (0.7–9.6 mm); however, the actual displacement ranged from −9.6 to + 5.4 mm (-, posterior; +, anterior). The absolute mediolateral displacement was 2.7 ± 3.4 mm (0.3–9.9 mm), and the actual displacement ranged from −9.9 to + 2.2 mm (-, medial; +, lateral).

The anteversion angle of the cup was 15.8 ± 15.4° (−4.2–27.5°), and the abduction angle was 47.7 ± 8.5° (35–60.8°).

Regarding complications, no deep infection or nerve injury were observed during the follow-up. One case of dislocation occurred within 6 weeks after surgery. Posterior dislocation of the hip occurred when the patient bent to pick up things, which caused extreme flexion of the hip joint. The anteversion angle of the acetabular cup was 24.2°, and the anteversion angle of the femur was 23.4° with an abduction angle of 47.6°. After successful closed reduction and 3 weeks’ rest on bed, no recurrent dislocation was observed during the follow-up. No obvious prosthesis loosening and periprosthetic osteolysis, according to our standards defined above, were noted radiographically during the follow-up. No re-revision was performed during our follow-up.

## Discussion

Tantalum trabecular augment combined with cup has been used in clinical practice for years and studies have proved its long-term prognosis (Whitehouse et al., 2015; Löchel et al., 2019). In the study by Whitehouse et al (Whitehouse et al., 2015), which had a minimum follow-up of 10 years, the overall survival was 92%, and at the last follow-up, a normal centre of rotation was restored in 90% of patients with a high hip rotation center preoperatively. Different sizes of augment can provide support for the acetabular cup in most bone defects (Issack, 2013; Ting-Xian et al., 2017). However, in some complex bone defects, it is difficult to accurately restore the hip rotation center using a conventional augment combined with an acetabular cup (Xiao et al., 2021). In addition, the variability in defect shape in patients with severe defects often leads to a poor fit of the augment to the defect. Customized 3D-printed prosthesis can precisely match the shape of bone defect, thus restoring the hip rotation center (Hughes et al., 2017). Dion et al. (2020) reported the application of 3D-printed augments in revision total knee arthroplasty and showed better fixture stability than that with conventional therapy. Fu et al. (2020) demonstrated the satisfactory biocompatibility and biomechanical features of a customized augment in a swine model with acetabular defect. Titanium is the most commonly used metal for 3D printing, and use of a 3D-printed customized titanium augment with a tantalum cup combines the bone ingrowth performance of tantalum with the ability of 3D-printed augments to restore the hip rotation center. Thus, all defects were effectively reconstructed in revision surgery based on intraoperative evaluation and satisfactory functional outcomes.

**FIGURE 4:**
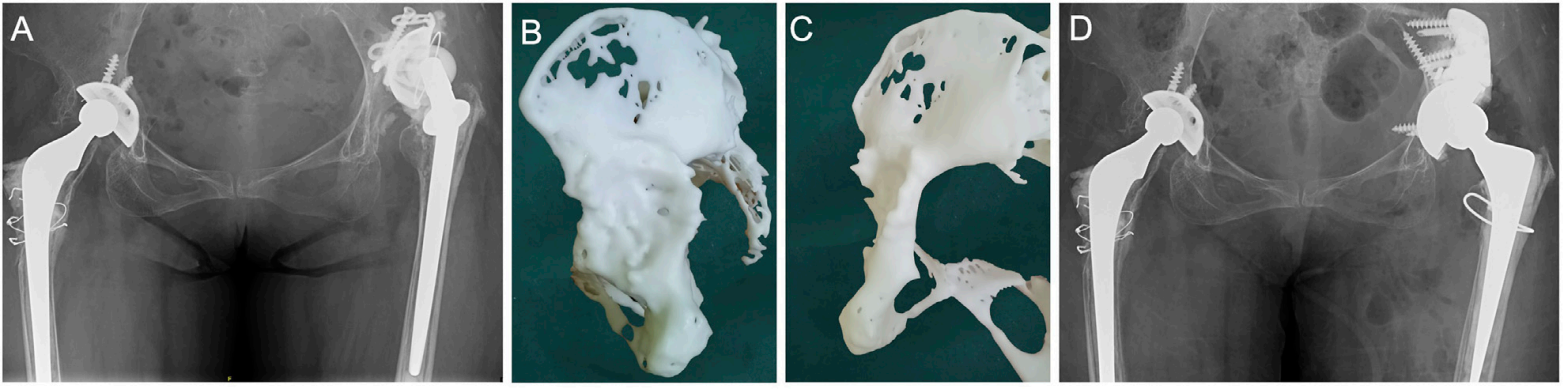
A representative patient receiving this combined therapy. **(A)** Preoperative AP pelvic x-ray of this patient. **(B,C)** Rapid prototype reconstruction of this patient’s acetabulum. **(D)** Postoperative AP pelvic x-ray taken 3 years after revision surgery. Reproduced with permission from authors (Zhang et al., 2022).

New technologies, such as 3D printing and RP, which have developed rapidly in the recent years (Hughes et al., 2017; Loganathan et al., 2020), help surgeons to visualize local bone defects, design customized implants, and permit preoperative simulation and surgical planning (Li et al., 2013; Li et al., 2016; Zhang et al., 2021). A trial installation is important, and surgeons should ensure the most effective position to stabilize the acetabular cup. A locking screw should be used first to fix the augment position. A representative case is shown in Figure 4. Thus, the amount of intraoperative bleeding and operation time can be significantly reduced (Hughes et al., 2017). In our study, RP was applied for designing and precise positioning of the augment during surgery. RP-assisted pre-operative planning could effectively guide augment implantation during revision THA, and RP-assisted implantation of augments greatly increased the accuracy of restoring centre of rotation (Xiao et al., 2021). In our study, a relatively normal hip rotation center was restored in all patients after surgery. Further, compared with the contralateral side, the hip rotation center was displaced upward by an average of 3.7 mm postoperatively. The average absolute anteroposterior and mediolateral displacement was 4.1 and 2.7 mm, respectively. Compared with those of other studies using conventional augments (Grappiolo et al., 2015; Xiao et al., 2021), our results show a more satisfactory restoration of the hip rotation center. Xiao et al. (2021) reported a wide variation in vertical hip centre distance ranging from 11.7 to 42.9 mm and a horizontal distance from 20.8 to 49.2 mm postoperatively. Moreover, two patients had a high hip rotation center. However, in revision THA in patients with severe bone defects, the placement of the cup is largely restricted by the quality of the residual bone since adequate initial stability is highly emphasized in revision surgery. The installation of a cage is less restricted due to the flexibility of the iliac wings. Therefore, there is some variation in the hip rotation center position and cup anteversion angle in our results. In this study, only one patient suffered a posterior dislocation during early rehabilitation. Since the 24.2° anteversion and 47.6° abduction angles of the cup are within the respective normal ranges, we believe this dislocation was not related to the anteversion and abduction of the cup. The scar tissues around the hip were widely excised during revision, which resulted in loose soft tissues around the prosthesis. Additionally, the dislocation occurred when the patient bent the body to pick up things. Thus, the dislocation probably occurred because of excessive flexion of the hip during movement.

Theoretically, wear and fretting corrosion will occur when different interface metals rub against each other in the electrolyte environment *in vivo*, especially in the assembled prosthesis (Brown et al., 2006). Thus, the friction between the titanium and cobalt-chromium alloy will produce metal ions. Back et al. (2005) reported that the levels of cobalt and chromium ions increased continuously in the first 6 months and remained high during a 2-years follow-up. The increase in local metal ion concentrations may cause osteolysis (Back et al., 2005; Delaunay et al., 2010). In our patients, friction between titanium and tantalum may lead to corrosion. However, no osteolysis was detected at the midterm follow-up, and no symptoms of metal ion accumulation such as local pain and inflammatory pseudotumor were noted (Liow and Kwon, 2017). This could be attributed to the fact that the tantalum cup and titanium augment are fixed using bone cement for less peri-implant stress shielding, which isolates the components. Moreover, tantalum is highly inert and relatively corrosion resistant (Levine B. R. et al., 2006).

Our study had some limitations. First, this was a retrospective study without a control group. Severe acetabular defects are challenging for most surgeons, and conventional augments cannot be used for complex cases; thus, it was difficult to define a control group. However, our findings provide sufficient evidence to prove the midterm effectiveness and safety of our approach. Second, due to the limited number of cases of severe acetabular defects treated in our institution, the number of cases included in this study was relatively small. Therefore, multi-centre studies with larger study cohorts are required in the future.

## Conclusion

Concurrent use of a 3D-printed augment and tantalum trabecular cup combines the advantages of both, such as the customization of 3D printing and biocompatibility and osteo-induction ability of tantalum. Using this strategy, we could effectively reconstruct severe acetabular defects during surgery Harris hip scores increased, and the survival rate was high with no revision at an average follow-up of 4.7 years.

## Data Availability

The raw data supporting the conclusions of this article will be made available by the authors, without undue reservation.
